# Opportunistic treatment of hepatitis C virus infection (OPPORTUNI-C): study protocol for a pragmatic stepped wedge cluster randomized trial of immediate versus outpatient treatment initiation among hospitalized people who inject drugs

**DOI:** 10.1186/s13063-020-04434-8

**Published:** 2020-06-15

**Authors:** H. Midgard, A. K. Finbråten, K. B. Malme, R. M. Berg-Pedersen, L. Tanum, I. C. Olsen, R. Bjørnestad, O. Dalgard

**Affiliations:** 1 Department of Infectious Diseases, Akershus University Hospital, Lørenskog, Norway; 2 Department of Gastroenterology, Oslo University Hospital, Oslo, Norway; 3 Department of Medicine, Lovisenberg Diaconal Hospital, Oslo, Norway; 4 Unger-Vetlesen Institute, Lovisenberg Diaconal Hospital, Oslo, Norway; 5 Department of Illicit drug use, Oslo University Hospital, Oslo, Norway; 6 Department for Research and Development in Mental Health, Akershus University Hospital, Nordbyhagen, Norway; 7 Oslo Metropolitan University, Oslo, Norway; 8 Department of Research Support for Clinical Trials, Oslo University Hospital, Oslo, Norway; 9 ProLAR Nett, Søgne, Norway; 10 Institute of clinical Medicine, University of Oslo, Oslo, Norway

**Keywords:** Hepatitis C virus, People who inject drugs, Direct-acting antivirals, Model of care, Pragmatic clinical trial, Stepped wedge cluster randomized trial, Study protocol, Reinfection, Resistance-associated substitutions

## Abstract

**Background:**

Scaled-up direct-acting antiviral (DAA) treatment of hepatitis C virus (HCV) infection among people who inject drugs (PWID) is crucial to reach the World Health Organization HCV elimination targets within 2030. One of the critical obstacles to HCV care in this population is the lack of treatment models within specialist healthcare adapted to marginalized individuals.

**Methods:**

OPPORTUNI-C is a pragmatic stepped wedge cluster randomized trial comparing the efficacy of immediate initiation of HCV treatment with the current standard of care among PWID admitted for inpatient care. Screening for HCV RNA will be performed as soon as possible after admission. The intervention includes immediate non-invasive liver disease assessment, counseling, and initiation of pan-genotypic DAA treatment with individualized follow-up. Standard of care is a referral to outpatient care at discharge. To mimic usual clinical practice as closely as possible, we will use a pragmatic clinical trial approach utilizing clinical infrastructure, broad eligibility criteria, flexible intervention delivery, clinically relevant outcomes, and collection of data readily available from the electronic patient files. The stepped wedge design involves a sequential rollout of the intervention over 16 months, in which seven participating clusters will be randomized from standard of care to intervention in a stepwise manner. Randomization will be stratified according to cluster size to keep high prevalence clusters separated. The trial will include approximately 220 HCV RNA positive individuals recruited from departments of internal medicine, addiction medicine, and psychiatry at Akershus University Hospital, Oslo University Hospital, and Lovisenberg Diaconal Hospital, Oslo, Norway. Individuals not able or willing to give informed consent and those with ongoing HCV assessment or treatment will be excluded. The primary outcome is treatment completion, defined as dispensing of the final prescribed DAA package from the pharmacy within 6 months after inclusion. Secondary outcomes include treatment uptake, virologic response, reinfection incidence, and resistance-associated substitutions.

**Discussion:**

Representing a novel model of care suited to reach and engage marginalized PWID in HCV care, this study will inform HCV elimination efforts locally and internationally. If the model proves efficacious and feasible, it should be considered for broader implementation, replacing the current standard of care.

**Trial registration:**

ClinicalTrials.gov, NCT04220645. Registered on 7 January 2020.

## Administrative information

Note: the numbers in curly brackets in this protocol refer to SPIRIT checklist item numbers. The order of the items has been modified to group similar items (see http://www.equator-network.org/reporting-guidelines/spirit-2013-statement-defining-standard-protocol-items-for-clinical-trials/).

**Table:** 

Title {1}	Opportunistic treatment of hepatitis C virus infection (OPPORTUNI-C): Study protocol for a pragmatic cluster randomised trial of immediate versus outpatient treatment initiation among hospitalized people who inject drugs
Trial registration {2a and 2b}	ClinicalTrials.gov NCT04220645. Registered on January 7 2020.
Protocol version {3}	Version 2. March 9 2020.
Funding {4}	The study was funded by a research grant from the Norwegian Health Trust Helse Sør-Øst. The funding body had no role in design of the study, collection, analysis, and interpretation of data, nor writing of the manuscript.
Author details {5a}	^1^Department of Infectious Diseases, Akershus University Hospital, Norway ^2^Department of Gastroenterology, Oslo University Hospital, Norway ^3^Department of Medicine, Lovisenberg Diaconal Hospital, Norway ^4^Unger-Vetlesen Institute, Lovisenberg Diaconal Hospital, Norway ^5^Department of Illicit drug use, Oslo University Hospital, Norway ^6^Department for Research and Development in Mental Health, Akershus University Hospital, Norway ^7^Oslo Metropolitan University, Norway ^8^Department of Research Support for Clinical Trials, Oslo University Hospital, Norway ^9^ProLAR Nett, Søgne, Norway ^10^Institute of clinical Medicine, University of Oslo, Norway
Name and contact information for the trial sponsor {5b}	Helge Røsjø, Department of research, Akershus University Hospital, 1478 Lørenskog, Norway.
Role of sponsor {5c}	The sponsor played no part in study design; collection, management, analysis, and interpretation of data; writing of the report; and the decision to submit the report for publication.

## Introduction

### Background and rationale {6a}

Globally, 70 million people are living with chronic hepatitis C virus (HCV) infection [1]. The mortality of HCV-related liver disease is considerable due to liver cirrhosis complications, including hepatocellular carcinoma [2, 3]. In Western Europe, two-thirds of the HCV disease burden is attributable to injecting drug use [4] and transmission continues to occur among people who inject drugs (PWID) [5]. In Norway, approximately 15,000 individuals were living with chronic HCV infection in 2017. Since then, direct-acting antiviral (DAA) HCV treatment has become broadly available; by early 2020, the Norwegian HCV population counts approximately 6000 individuals (data on file), of whom > 80% are former or recent PWID [6].

The introduction of highly efficient DAA therapy has changed the HCV treatment paradigm, leading to cure in > 95% of individuals after an 8–12-week course of oral treatment [7]. Although DAA therapy is shown to be effective in clinical trials and cohort studies of selected PWID [8, 9], HCV care in this group may be challenging due to risks of suboptimal treatment adherence [10], loss to follow-up [9], and reinfection after successful treatment [11, 12]. Suboptimal adherence could increase the risk of selection of resistance-associated substitutions in the viral genome [13], but the clinical importance of this among PWID is unknown. Improving HCV treatment uptake among PWID will be crucial to achieving the World Health Organization goal of eliminating HCV infection as a major public health threat before 2030 [1]. There is therefore a need for more generalizable data on successful models of care and treatment outcomes, including the magnitude of reinfection and resistance, particularly among more marginalized PWID [14, 15].

One of the critical obstacles to HCV care in this population is the lack of treatment models within specialist healthcare adapted to marginalized individuals [15–17]. The current standard of care, involving referral of patients to specialist care at hospital outpatient clinics, has proven ineffective in this group due to low attendance and lack of retention in the care cascade [18–21]. Even low-threshold outreach clinics may be unable to reach the most marginalized individuals [22], e.g. younger amphetamine users not engaged in opioid agonist therapy with only sporadic contacts with healthcare services. However, this population is at risk of emergency hospitalization for inpatient treatment of various conditions, such as drug-dependency disorders, intoxications, psychosis, soft-tissue infections, deep venous thrombosis, endocarditis, and liver disease. We hypothesize that such hospitalizations represent opportunities to engage marginalized PWID in HCV care more effectively than the current standard of care.

OPPORTUNI-C will assess the efficacy of a novel opportunistic model of care, providing immediate HCV assessment and treatment for hospitalized PWID in a pragmatic clinical trial with a stepped wedge cluster randomized trial design. Despite clear evidence of the efficacy of DAA treatment among PWID [9] and preliminary evidence supporting the opportunistic approach [23], no controlled study has to date evaluated the efficacy of similar interventions at the level of health service delivery.

## Objectives {7}

The primary objective of OPPORTUNI-C is to evaluate the feasibility and efficacy of an opportunistic treatment strategy with immediate assessment and treatment of HCV infection among PWID admitted for inpatient care in departments for internal medicine, addiction medicine, and psychiatry.

Secondary/exploratory objectives are the following:

- to calculate the incidence of HCV reinfection following successful treatment among all study participants;
- to assess the frequency of resistance-associated substitutions at baseline among all participants, and during treatment among those with virologic treatment failure;
- to compare HCV testing uptake among PWID admitted for inpatient care, HCV RNA prevalence among those tested, and HCV treatment uptake among HCV RNA positive individuals, before and after implementation of the trial.

## Trial design {8}

### Pragmatic clinical trial

To mimic usual clinical practice as closely as possible in order to capture a representative study population and generate optimal conditions for generalizability at a low cost, we will design the trial as pragmatic as possible. A pragmatic feature is to measure the effectiveness of the intervention in routine clinical practice in order to inform policy decision-makers. As such, we aim to provide evidence of immediate relevance to the decisions of patients, healthcare professionals, and policymakers by assessing interventions as prescribed, managed, and used in routine clinical practice [24]. For OPPORTUNI-C, we will apply the following characteristics:

- recruitment from a clinical setting that will capture the target population relatively unbiased;
- broad eligibility criteria that will exclude few participants;
- clinical infrastructure with minimal separately constructed research structure;
- flexible intervention delivery compatible with routine clinical practice;
- clinically relevant and easily accessible outcome measures;
- extraction of routinely collected data readily available from the electronic patient files and local microbiological databases, making individual follow-up for data collection unnecessary;
- data analysis according to an intention-to-treat principle.

### Stepped wedge cluster randomized trial design

**Fig. 1:**
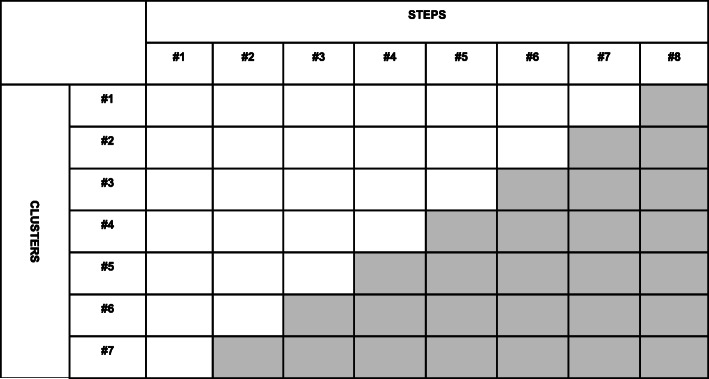
The stepped wedge cluster randomized trial design of OPPORTUNI-C. Seven clusters will be sequentially assigned to change from standard of care to intervention in a random order. Blank cells represent control observations and shaded cells represent intervention observations. The length of each step is 2 months

OPPORTUNI-C is a multicenter study utilizing a stepped wedge cluster randomized trial design [25]. Participants will be recruited from seven departments of internal medicine, addiction medicine, and psychiatry at three hospitals in Oslo and Akershus counties, Norway. The design involves a sequential rollout of the intervention, in which the seven participating clusters will be randomized from standard of care to an opportunistic treatment strategy in a stepwise manner over 16 months. The length of each step will be 2 months and one cluster will be randomized at each step. A schematic presentation of the stepped wedge design is shown in Fig. 1.

In order to evaluate the effect of a complex intervention at the level of health service delivery, cluster randomization at the department level instead of individual randomization at the patient level will be performed. Further, cluster randomization will eliminate the risk of “contamination” or spill-over of the intervention between local healthcare providers or study participants.

The rationale for choosing a stepped wedge design over a traditional parallel cluster design rests on the following logistical, political, and statistical considerations:

- a sequential rollout is preferable for logistical reasons, enabling a gradual implementation of the intervention across several heterogeneous centers;
- assuming a superiority of the intervention, there would be considerable difficulties recruiting clusters to a parallel design in which only half of the clusters would receive the intervention;
- it is likely to be a more efficient and robust design due to an anticipated sizeable intra-cluster correlation and relatively large cluster sizes.

## Methods: participants, interventions, and outcomes

### Study setting {9}

#### Participating clusters

Participants will be recruited from the following seven clusters:

- Department of Illicit Drug Use, Akershus University Hospital
- Department of Psychiatry, Acute Ward, Akershus University Hospital
- Clinic of Medicine, Akershus University Hospital
- Department of Illicit Drug Use, Acute Ward, Oslo University Hospital
- Clinic of Medicine, Oslo University Hospital
- Department of Psychiatry, Lovisenberg Diaconal Hospital
- Department of Medicine, Lovisenberg Diaconal Hospital

There were no strict pre-specified cluster eligibility criteria, but the selection was based on clinical experience that individuals with HCV infection are well represented in these departments. Representing all emergency hospitalizations in internal medicine, addiction medicine, and psychiatry at the three major hospitals in the larger Oslo area, the participating clusters will collectively cover a population of approximately one million individuals and potentially capture a high proportion of those at risk of HCV infection residing in the area. All departments who were approached agreed to participate.

### Eligibility criteria {10}

#### Patient inclusion criteria

The participants must meet all of the following criteria:

- age ≥ 18 years
- HCV RNA positive
- admitted for inpatient care at a participating department
- signed informed consent obtained according to national and local regulations

#### Patient exclusion criteria

Individuals will be excluded from participation if they meet any of the following criteria:

- pregnancy or breastfeeding
- ongoing assessment or treatment of HCV infection
- current participation in another trial that might affect the current study
- unable or unwilling to give informed consent for any reason

Ongoing assessment is defined as already initiated pre-treatment work-up in the outpatient setting, i.e. performed virologic assessments or liver disease staging with the intention to initiate treatment. Individuals who have been referred to outpatient care but not (yet) met for consultation, or individuals considered to have dropped out of care, will not be excluded*.* Participants can withdraw from the study at any time without any rationale and without compromising their future medical care.

#### Screening for HCV RNA

Following usual practice, HCV RNA testing will be performed on clinical indication as soon as practically possible after admission, using a conventional qualitative in-house PCR assay. HCV RNA will be analyzed on all weekdays at Oslo University Hospital and Lovisenberg Diaconal Hospital and twice weekly at Akershus University Hospital. When a hospitalized patient is found to be positive for HCV RNA, the local microbiological departments will immediately alert the local investigator by telephone to expedite patient inclusion.

In the two departments of addiction medicine, prevalence of HCV RNA is expected to be relatively high. Therefore, all individuals will be screened for HCV RNA at admission and informed consent will be obtained in the testing situation. In the remaining five clusters, testing for HCV RNA will be performed only in the presence of risk factors for HCV transmission and informed consent will generally be obtained only in individuals with detectable HCV RNA. One exception will be the Department of Psychiatry at Akershus University Hospital, where screening for anti-HCV antibodies will be performed among all individuals at admission.

### Who will take informed consent? {26a}

An investigator at each study location will be engaged to obtain informed consent and facilitate participant enrolment, but a trained delegate can also do this. The process of informed consent will include the delivery of balanced written information and by a discussion concerning the need and overall benefit of the trial. This discussion will include a check of understanding concerning the benefits and risks of participation and ensuring that participants accept that the intervention will be allocated at random regardless of any personal preference they may have. The consent form includes contact information for the primary investigators, providing opportunities for study participants to discuss further details later.

The consent form includes a simplified and easily comprehensible summary the study rationale and study design, including procedures for control and intervention periods. This is followed by a section describing possible benefits (i.e. contribution to knowledge production) and harms (i.e. potential adverse events [AEs] of DAAs) associated with participation and a section covering the voluntary nature of participation and the option to withdraw from the study after inclusion. The next sections describe the routines for data management and confidentiality (including post-treatment follow-up and biobanking), insurance (covered by the Norwegian System of Patient Injury Compensation), and economy (study funding and reimbursement of DAA treatment). The final part provides the ethics approvals of the study and contact information for the local primary investigators and data protection officials, followed by a section for signatures from the participant and study personnel.

### Additional consent provisions for collection and use of participant data and biological specimens {26b}

Given that HCV RNA is not analyzed daily in any cluster and the expected time lag from a blood sample is drawn to the investigator is alerted, we expect to see that a significant number of eligible patients are discharged before informed consent has been collected. These individuals will be informed in writing that restricted data will be collected retrospectively for a dropout analysis (sensitivity analysis) unless an explicit reservation is obtained within 6 weeks.

## Interventions

### Explanation for the choice of comparators {6b}

The comparator is the referral of HCV RNA positive individuals for outpatient HCV care after discharge. Although other models of HCV care have been successfully implemented in other healthcare settings for PWID, an outpatient referral is widely considered as the standard of care for HCV infection among hospitalized individuals. To the best of our knowledge, immediate treatment initiation among hospitalized individuals is only rarely performed and under special circumstances.

### Intervention description {11a}

#### Both trial arms

**Fig. 2:**
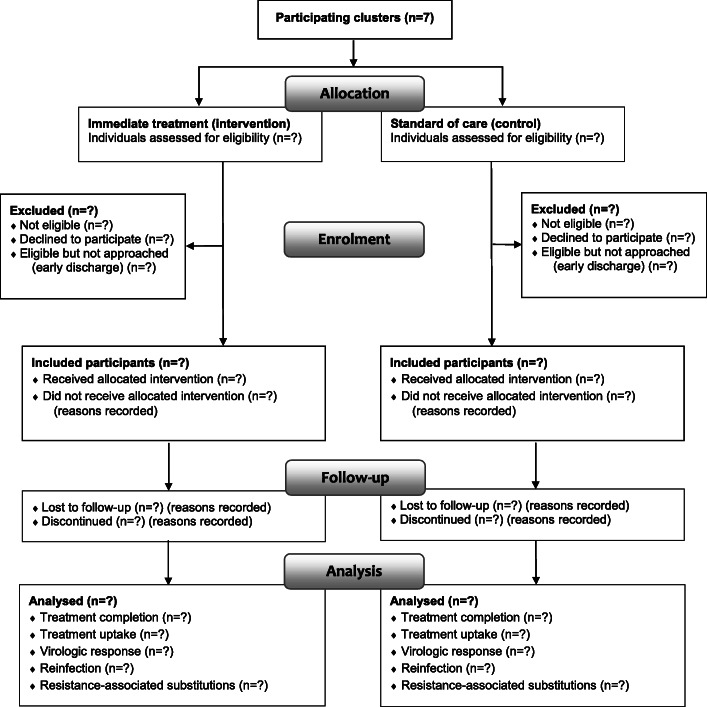
CONSORT *diagram* showing flow of study participants in OPPORTUNI-C

Both during control and intervention periods, testing for HCV RNA will be performed as soon as possible after admission, as described earlier. Before and during the study period, nurses and physicians at all participating wards will be given lectures presenting key aspects of HCV epidemiology and care as well as specific information about the present trial. Information will also be disseminated via e-mail, newsletters, flyers, and posters summarizing the study concept will be distributed to all departments. A CONSORT diagram of patient flow is shown in Fig. 2.

#### Control group

During control periods, all included participants will be offered standard of care, which includes referral of HCV RNA positive individuals for outpatient HCV care after discharge. Immediate staging of liver disease and pre-treatment counseling is not considered a part of standard of care and will usually not be performed. Local practice will decide whether it is the outpatient clinic for infectious diseases or for gastroenterology that will be responsible for further assessment and treatment after discharge. Individuals with multiple admissions will be given care according to the period in which they originally were included; reinclusion will not be allowed.

#### Intervention group

Following cluster allocation from standard of care to intervention, individuals positive for HCV RNA will be offered immediate initiation of HCV treatment during hospitalization, at discharge, or as soon as possible after discharge. The intervention includes the following four components:

1. *Liver disease staging*

Measurements of liver stiffness with transient elastography using FibroScan® will be performed in all participants positive for HCV RNA by a trained operator, usually the local primary investigator or a trained nurse. Transient elastography is available at all study locations except at the Department of Illicit Drug Use at Oslo University Hospital, where the stage of liver fibrosis will be assessed with the serum fibrosis markers AST-to-platelet ratio index-score and Fibrosis-4 index. An ambulant transient elastography service will be available for this department on request. All participants with suspected liver cirrhosis will be scored according to the Child Pugh classification.

2. *Pre-treatment counseling*

All participants will be given counseling at the discretion of the treating physician or nurse in teamwork with the local primary investigator. Standard counseling includes disclosure of HCV RNA status, stage of liver disease and prognosis, potential co-infections, as well as information regarding potential DAA treatment AEs, drug–drug interactions, and requirements for follow-up. All patients will be advised on measures to minimize reinfection risk and other harms associated with injecting drug use.

3. *Treatment initiation*

All included individuals will be offered to initiate HCV treatment as soon as possible during hospitalization, at discharge, or as soon as possible after discharge. Treatment will be prescribed in accordance with current HCV Norwegian and European treatment recommendations. Currently, first-line Norwegian DAA recommendations include sofosbuvir/ledispavir for 8 weeks for genotype 1 infection without liver cirrhosis and sofosbuvir/velpatasvir for 12 weeks for genotype 2 or 3 infection. However, HCV genotype will rarely be available during the hospital stay, as this analysis typically is performed only once every second week. Thus, in order to ensure rapid treatment initiation and minimize loss to follow-up, pan-genotypic DAA treatment with sofosbuvir/velpatasvir for 12 weeks or glecaprevir/pibrentasvir for 8 weeks will be the preferred regimens for most participants. The choice of DAA regimen will be at the discretion of the prescriber.

Normally, local consultants of infectious diseases or gastroenterology will prescribe HCV treatment for participants admitted to the internal medicine and psychiatry departments, while we will encourage local addiction medicine physicians to prescribe treatment for those admitted for addiction care. In many cases, the local primary investigator will prescribe treatment. We expect that, over time, local physicians will grow increasingly clinically independent in the field of HCV care, reflecting an educational aspect of the trial.

DAA treatment will be prescribed using an electronic prescription (“H-prescription”) developed for drugs purchased at a discounted price by the Norwegian Health Trusts. A nurse will have the possibility to collect the first DAA package from the hospital pharmacy, ensuring prompt treatment initiation. Prescriptions will be re-iterated to include the complete treatment course. However, to minimize the risk of potential illegal trade of medications after discharge, only one DAA package should be dispensed at a time. This will also allow assessment of the primary outcome.

4. *Individualized follow-up*

The intensity of care after discharge will be individualized at the discretion of the treating physician. Many participants will be expected to administer the 8–12 weeks treatment without assistance, but some will require close follow-up with the aid from local low-threshold services, the local outpatient clinic or the general practitioner. On-treatment follow-up with HCV RNA monitoring at treatment week 4, end of treatment (treatment week 8 or 12), and 12 weeks after treatment is recommended in line with usual practice. No prospective data collection will be performed following discharge, as assessments primarily will be made by registry linkage. Post-treatment follow-up (e.g. hepatocellular carcinoma surveillance for individuals with liver cirrhosis) will be provided in accordance with usual care.

### Criteria for discontinuing or modifying allocated interventions {11b}

There are no criteria for discontinuing or modifying allocated interventions except what is recommended usual practice. For instance, DAA treatment may be discontinued in response to a worsened liver function, particularly regimens containing a protease inhibitor (see “Adverse event reporting and harms” below). Participants may choose to discontinue DAA treatment themselves for any reason.

### Strategies to improve adherence to interventions {11c}

There will be no particular strategies beyond standard encouragement, but HCV RNA monitoring, as described above, will be recommended in line with current guidelines. In accordance with a pragmatic approach, economic incentives, or electronic blister packs for monitoring of tablet intake, will not be offered to improve treatment adherence. However, for some individuals, direct-observed therapy may be initiated at the discretion of the treating physician.

### Relevant concomitant care permitted or prohibited during the trial {11d}

There are no restrictions beyond recommended usual care. For instance, potential drug–drug interactions will be checked before treatment initiation.

### Provisions for post-trial care {30}

All participants with suspected liver cirrhosis will be offered post-treatment hepatocellular carcinoma surveillance with ultrasound and measurement of serum alpha-fetoprotein every 6 months according to international guidelines. Furthermore, all participants with ongoing injecting drug use will be advised to seek testing for HCV RNA annually or biannually after successful treatment in line with international recommendations.

### Outcomes {12}

All outcomes will be assessed by review of the electronic patient files and local microbiological databases after inclusion of the final participant. In our opinion, the outcomes harmonize well with a pragmatic approach, as they are clinically relevant and correspond to standard clinical metrics that are easy to collect and analyze retrospectively without the need for a separate research infrastructure for follow-up.

#### Primary outcome

The primary outcome is treatment completion. The primary outcome is denoted as a success if the participant has been dispensed the final 4-week package of the prescribed DAAs within 6 months after inclusion, and failure otherwise. The final package is defined as the second package in individuals receiving 8 weeks of treatment regimens (sofosbuvir/ledipasvir or glecaprevir/pibrentasvir) and the third package in individuals receiving 12 weeks of treatment regimens (sofosbuvir/velpatasvir).

The primary outcome will be assessed at 6 months after inclusion of the final participant by review of the prescription module (“Reseptformidleren”) and the “core medical record “(“Kjernejournal”) in the hospital electronic patient files. Collectively, these sources will capture all prescriptions nationwide and also provide data on the prescriber, the number of dispensed packages, and the date of distribution. The primary outcome is pragmatic in terms of being a proxy for completion of treatment instead of a measure of self-reported or actual adherence to the prescribed DAAs. In our opinion, this outcome corresponds well to an increasingly employed clinical metric in the DAA treatment era, reflecting improved treatment uptake among more marginalized individuals at risk of loss to follow-up.

#### Secondary outcomes

- *Treatment uptake*, defined as having been dispensed at least one package of DAAs within 6 months after inclusion. This outcome will be assessed at 6 months after inclusion of the final participant by review of the prescription modules as described above.
- *Virologic response*, defined as undetectable HCV RNA in any available blood sample drawn as close to the estimated time of treatment completion as possible, at least 8–12 weeks after distribution of the first DAA package, depending on the regimen. Participants who have not been dispensed any DAAs, those with no blood samples available, or those with a HCV RNA positive sample following an initial virologic response, will be characterized as treatment failures. This outcome will be assessed 6 months after inclusion of the final participant by review of local microbiological and clinical databases. Being a proxy for actual virologic cure, undetectable HCV RNA will not necessarily correspond to a sustained virologic response, although it will represent an end of treatment response in most cases. As discrepancies between end of treatment and sustained virologic response are assumed negligible, we consider this a pragmatic, feasible, and valid outcome for the DAA era.

#### Virological outcomes

The following outcomes are not effect measures, but purely observational:

- *Reinfection*, pragmatically defined as recurrence of HCV RNA in any available blood sample after a documented virologic response. This outcome will be assessed at 12 months after inclusion of the final participant by review of local microbiological databases. In addition, a more stringent reinfection diagnosis will be made based on the presence of a HCV strain not present at baseline in individuals with HCV RNA recurrence following a virologic response. This will be assessed comparing prospectively collected recurrent samples with stored baseline samples using whole genome deep sequencing methods, as described later.
- *Resistance-associated substitutions*, defined as presence of a clinically important resistance-associated substitution before initiation of treatment or emergence of a clinically important resistance-associated substitution at the time of a virologic treatment failure. This will be assessed using whole genome deep sequencing of HCV in (1) stored baseline samples and in (2) prospectively collected samples demonstrating HCV RNA breakthrough during treatment or recurrence of HCV RNA between end of treatment and virologic response.

#### Epidemiological outcomes

The following observations will also be assessed:

- *HCV testing uptake*, defined as the proportion of hospitalized PWID having been tested for HCV RNA, assessed by review of local microbiological and clinical databases. PWID will be identified by searching the administrative systems for relevant ICD-diagnoses associated with drug intoxications, harmful drug use, and drug dependency (F10-F19) among all individuals admitted for inpatient care in all participating clusters. Uptake of testing during the study period will be compared to uptake of testing during a historical control period before the implementation of the study. This will be performed in a dedicated sub-study defined as a quality improvement project without the requirement for informed consent. A separate protocol for this sub-study is under development.
- *HCV RNA prevalence*, defined as the proportion of tested PWID with detectable HCV RNA, assessed by review of local microbiological and clinical databases. Uptake of treatment during a historical control period before the implementation of the study will then be compared to uptake of treatment during the study period, as described above. This will also be performed in a dedicated sub-study (see above).

### Participant timeline {13}

**Fig. 3:**
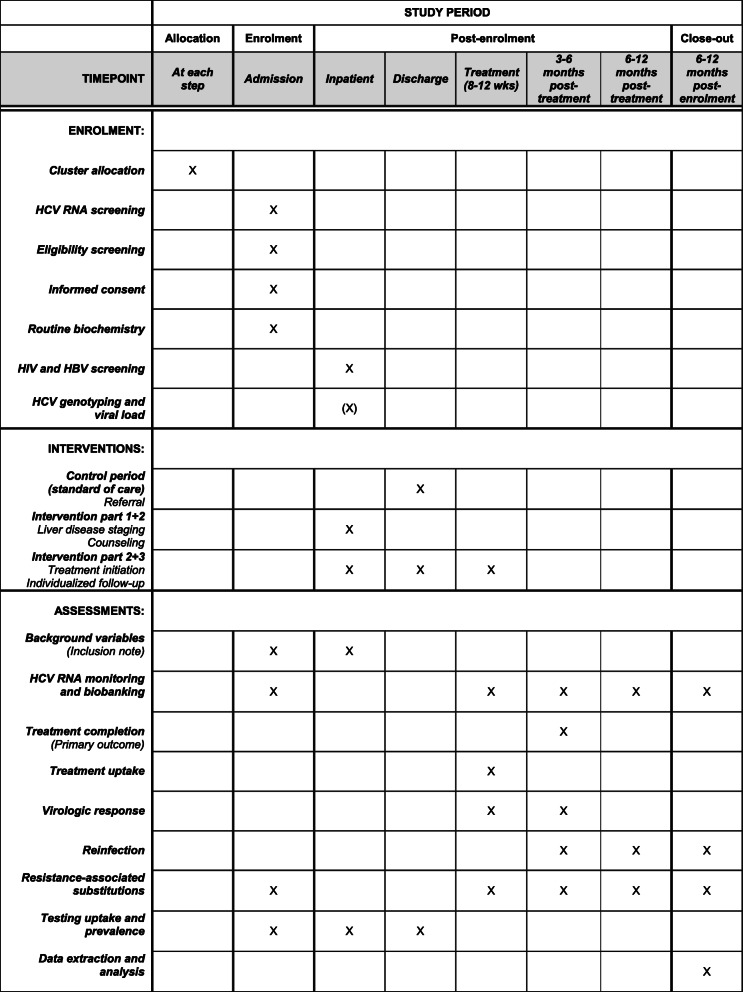
Schedule of enrolment, interventions, assessments, and visits for participants in OPPORTUNI-C

A schedule of enrolment, interventions, assessments, and visits for participants is shown in Fig. 3.

### Sample size {14}

**Fig. 4:**
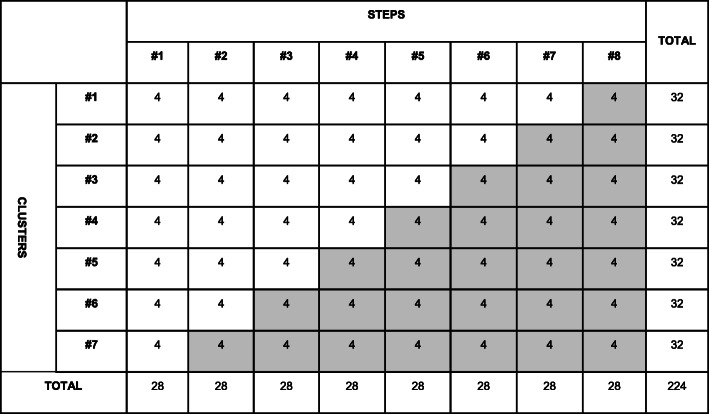
*Diagram* showing the estimated average number of included participants needed per cluster for each step of the study. *Shaded cells* represent intervention periods

Statistical power analysis was performed in STATA 15 using the stepped wedge procedure. In order to show a 30% difference in effect size (60% the intervention group vs 30% in the control group) for the primary outcome, with 85% power and 5% significance level, assuming a large intra-cluster correlation coefficient of 0.2, we will need, on average, four participants per cluster per 2-month step for a total of 224 included individuals during the study (Fig. 4). The average cluster size will be 32 participants, but there will be no absolute restriction on the number of participants per cluster.

As there are no published studies available to directly inform sample size calculations, assumptions of effects sizes and intra-cluster correlation are conservative, and based on our own clinical experience and data from clinical databases on Akershus University Hospital (data on file) and a low-threshold HCV clinic in downtown Oslo [22]. However, a sensitivity analysis including a wider range of estimates for effect size and intra-cluster correlation coefficient will be performed before data extraction and analysis. The stepped wedge design is considered appropriate and effective in situations with an expected large intra-cluster correlation, as it retains the statistical power available compared to alternative designs [26].

### Recruitment {15}

The key to achieving adequate and unbiased participant enrolment and reaching the target sample size will be that the vast majority of patients at risk of HCV infection are screened for HCV RNA at admission and that most eligible participants undergo a process of informed consent. Therefore, a local investigator at each study location will be engaged to facilitate the collection of informed consent and enrolment. If we suspect that an insufficient number of participants will be included, we will consider either prolonging the duration of the middle (fifth) step or increasing the length of each step from 2 months to 3 months. Eligible participants discharged before informed consent has been obtained will be informed in writing and given the opportunity for reservation before inclusion for a dropout (sensitivity) analysis, as described earlier.

## Assignment of interventions: allocation

### Sequence generation {16a}

Allocation is computer-generated and will be stratified according to cluster size to keep high prevalence clusters separated with regards to the timing of the intervention. The departments of internal medicine, addiction medicine, and psychiatry are considered large (strata 1), medium (strata 2), and small (strata 3), respectively. Thus, the departments in strata 1 with will be allocated to clusters 1, 4, or 7, the departments in strata 2 will be allocated to clusters 2 or 6, while the departments in strata 2 will be allocated to clusters 3 or 5 (Fig. 1). This stratified randomization process will prevent situations where for instance the three large departments are allocated to clusters 1, 2, and 3, thereby contributing to a high proportion of control observations and relatively little intervention observations.

### Concealment mechanism {16b}

The allocation sequence will be made available to the researchers in sequentially numbered, sealed, opaque envelopes on the day of transition to a new step of the trial.

### Implementation {16c}

The allocation sequence will be generated and prepared by a statistician not involved in patient recruitment. Participants will be enrolled by the local investigator or a trained delegate at each location.

## Assignment of interventions: Blinding

### Who will be blinded {17a}

Due to the nature of the study design, the delivery of the intervention is not blinded. The result of the concealment of a new step in the allocation sequence will be disclosed to patients, nurses and physicians at the relevant ward as soon as possible on the day of transition. Those involved in the data analyses and statistics will be blinded to the group allocation.

### Procedure for unblinding if needed {17b}

Study participants and clinical staff are not blinded.

## Data collection and management

### Plans for assessment and collection of outcomes {18a}

#### Data collection

In line with the pragmatic approach, we will collect data that are part of routine clinical practice already recorded at the hospitals for assessment of the primary and secondary outcomes. Data on sociodemographics, staging of liver disease, and biochemistry will be extracted from the electronic patient files. Virologic data will be retrieved from the patient files and clinical databases at the microbiological departments. Data on DAA prescriptions will be assessed by review of the prescription module “Reseptformidleren” and the “core medical record “(“Kjernejournal”) in the hospital electronic patient files.

A conventional case report form will not be used; however, to avoid missing key information, data on sociodemographics, clinical status, treatment plan, and cluster allocation will be summarized in a standardized journal note at the date of enrolment. Although these data will be collected and recorded at the time of enrolment, thus representing a “pseudo-case report form,” all data will be extracted from the electronic patient files retrospectively after completion of the study period. Potential entry errors in the standardized journal note will be controlled against the electronic patient files at the time of data extraction. The schedule of enrolment, interventions, and assessments is provided in Fig. 3.

#### Background variables

These variables will be limited to data characterizing the study population, including factors previously shown or hypothesized to be associated with the outcomes. The following sociodemographic background variables will be collected:

- age and gender
- history of injecting drug use
- recent (past 3 months) injecting drug use
- main type of drugs injected (opiates vs stimulants)
- recent (past 3 months) sharing of injecting equipment
- opioid agonist therapy status
- housing status
- employment status
- ethnic origin
- area of residence

The following clinical background variables will be collected:

- stage of liver disease (liver stiffness, AST-to-platelet ratio index, Fibrosis-4 index, and Child Pugh score if applicable)
- renal function (glomerular filtration rate)
- virology (HIV serology, HBV serology, HCV genotype, and HCV viral load, if available)
- discharge diagnoses

#### Prospective data collection for virological outcomes

Treated individuals will normally be scheduled for post-treatment follow-up for assessment of sustained virologic response in line with usual practice. However, as clinical practice may vary, and to prevent loss to follow-up, all individuals who fulfil the primary outcome (treatment completion) will be identified through the electronic patient files and offered a follow-up consultation (either ambulant or at the outpatient clinic) or a telephone interview. Individuals with a documented virologic response as defined earlier will be eligible for follow-up at regular (3–6-month) intervals. Assessments during follow-up will include testing for HCV RNA and a self-reported questionnaire collecting data on recent injecting drug use, recent injecting risk behaviors (e.g. frequency of injections and sharing of injecting equipment), opioid agonist therapy status, housing status, and employment status. This prospective data collection will be coordinated by the primary investigator and will involve a small economic incentive to encourage retention of participants.

#### Molecular analysis for virological outcomes

The analysis of resistance-associated substitutions and reinfection will be conducted using whole genome sequencing at the Norwegian Institute of Public Health. Whole genome sequencing is a highly sensitive technology that can provide a full characterization of the whole viral population, including resistance-associated substitutions independent of genotype and treatment regimen. The assay is designed to detect all HCV genotypes and sub-genotypes using the HCV-specific sequence library bait-enrichment approach combined with sequencing using the Illumina MiSeq-platform. Sequence data will first be quality checked and trimmed, followed by an initial reference-based mapping against a reference database, including all currently recognized genotypes and sub-genotypes. A consecutive reference-based mapping and whole genome assembly will follow verifying the correct sub-genotype. Resistance-associated substitutions will be determined in NS3, NS5, and NS5B by using the HCV-GLUE platform and linked to possible failed DAA treatment regimen.

Phylogenetic analyses will be performed based on maximum likelihood and/or Bayesian approaches, where virologic relapses will be distinguished from reinfections through the differential association of post-treatment virus (variant) sequences to either (I) a virus (variant) determined at baseline or (II) a novel virus (variant). Phylogenetic robustness will be assessed using bootstrap values. It can be challenging to distinguish closely related variants within a sub-genotype, as HCV is highly diverse, producing thousands of variants within the host that change over time. If we cannot distinguish HCV variants using the protocol outlined above, other techniques using long-reads on MinIon to characterize the virus on a single virus level may be applied to have significant power to distinguish viral variants that have emerged over a long course of infection [27]. In most cases of HCV RNA recurrence, a pragmatic reinfection diagnosis can be made in the absence of whole genome sequencing.

### Plans to promote retention of participants and complete follow-up {18b}

Nothing beyond normal encouragement will be done to promote retention of participants in the main trial (primary and secondary outcomes). However, to optimize prospective data collection for virological outcomes, a small economic incentive will be offered study participants to encourage retention at follow-up.

### Data management {19}

All participants will be given an individual subject number at enrolment, which will be noted in the standardized inclusion note in the electronic files. A code list linking the subject number to the personal identification number will be kept in a secure place at each study location. Data extraction from the electronic files and data entry into a secure trial database will be performed retrospectively 6 months after enrolment of the final participant. Double entry of data into the trial database will be performed.

### Confidentiality {27}

All collected information will be kept strictly confidential and stored in accordance with national regulatory approvals. The investigator at each site will be the only study personnel with access to the code list.

### Plans for collection, laboratory evaluation, and storage of biological specimens for genetic or molecular analysis in this trial/future use {33}

In order to perform whole genome sequencing, viremic sera drawn at baseline or during follow-up will be stored at – 70 °C in a study-specific biobank. This sequencing method requires analysis of 1 mL whole blood serum or plasma. We will strive to not take a new blood sample for the biobank, but to obtain the necessary serum or plasma from samples already drawn. Samples collected from the study participants will be shipped from the local microbiology departments to the central processing facility at the Norwegian Institute of Public Health for sequencing analysis, but final storage will be at Akershus University Hospital.

## Statistical methods

### Statistical methods for primary and secondary outcomes {20a}

A detailed plan for analysis will be developed before data extraction, but some of the principles of data analysis are outlined here. Analysis will be reported in accordance with the Consolidated Standards of Reporting Trials (CONSORT) statement and the suggested modifications to the CONSORT 2010 cluster extension for reporting of stepped wedge cluster randomized trials [28].

All trial data will be summarized by treatment group, cluster, and total. Continuous data will be reported as mean (SD), categorical data as N (%). Clusters will be analyzed according to their randomization regardless of what occurred, following an intention-to-treat principle. As time is an important potential confounder associated with the exposure to the intervention, it will be adjusted for in the analysis. To analyze the binary outcomes, we plan to use a mixed logistic regression model with a random effect for cluster and a fixed effect, including time, for each step in the trial. With the stepped wedge design, we can examine the impact of the intervention over time and include the time the cluster has been exposed to the intervention as an effect modifier. Moreover, with this design we can compare exposed and unexposed periods within a cluster, allowing exploration of heterogeneity in treatment effects between clusters. A mixed logistic regression model is considered appropriate and recommended for this design [25].

The null hypothesis that there is no effect of the intervention will be tested using the fixed step effect parameter estimate in the mixed logistic regression model. Superiority of the intervention over the control will be claimed if the *p* value under the null hypothesis, calculated by the Wald method, is < 0.05 (two-sided) in favor of the intervention. There will only be one primary analysis (one *p* value presented) and all secondary analyses will be presented with point estimates and confidence intervals only.

### Methods for additional analyses (e.g. subgroup analyses) {20b}

Subgroup analyses and adjusted analyses according to relevant background variables will be prespecified in the statistical analysis plan in a blinded manner. Additional analyses of the primary and secondary outcomes using time-to-event analysis will also be performed. The clinical rationale for this approach rests upon the notion that the intervention may be considered more clinically important the sooner it leads to treatment, reducing the duration of the viremia in the individual, and hence the duration of potential onward transmission.

Rates of reinfection incidence will be calculated using person-time techniques, assuming a Poisson distribution, as the number of participants with reinfection per 100 person-years of observation. The time of risk of reinfection will be calculated from the time of virologic response until the last available negative HCV RNA test for individuals without reinfection, and from the time of virologic response until the midpoint between the last negative HCV RNA test and the first positive HCV RNA test for individuals with reinfection. Factors associated with reinfection will be analyzed using Poisson regression.

### Methods in analysis to handle protocol non-adherence and any statistical methods to handle missing data {20c}

The primary analysis will follow an intention-to-treat principle with no account taken of protocol non-adherence. An additional analysis will be performed where patients are analyzed as treated, not as randomized. This will also include an analysis incorporating if treatment was initiated as planned initially, i.e. in the outpatient clinic for control observations, or during hospitalization for intervention observations.

Missing data for the primary endpoint will be regarded as failure. If, however, missing data are regarded as having a significant effect on the conclusions of the trial, sensitivity analyses with different methods for handling missing data will be included. Such methods may include complete case analyses, last observation carried forward, worst case/best case imputation, and multiple imputation techniques. The choice of missing data handling will be further specified in the statistical analysis plan. In order to explore the selection effect that may have been introduced by the informed consent procedure, an important sensitivity analysis will include the “dropout population” consisting of all eligible individuals positive for HCV RNA who were discharged before informed consent had been collected.

## Oversight and monitoring

### Composition of the coordinating center and trial steering committee {5d}

The trial will be overseen by a small project management group at the coordinating center, supported by a multidisciplinary trial steering committee. The project management group will consist of the project leader and the local primary investigators and will have meetings approximately every second week during the study period. The group will monitor trial progress and recruitment and be responsible for key decision-making. The trial steering committee will consist of members with complementary clinical and academic backgrounds from infectious diseases, hepatology, psychiatry, addiction medicine, microbiology, and medical statistics. The steering committee will also have a representative from the user organization proLAR, representing PWID receiving opioid agonist therapy in Norway. In our opinion, the user perspective is fundamental in order to develop feasible models of care that can inform system-level strategies for HCV elimination. Four trial steering committee meetings are planned.

### Composition of the data monitoring committee, its role, and reporting structure {21a}

A data monitoring committee will oversee trial safety and report to the project management group and the trial steering committee. The data monitoring committee will consist of one statistician and two physicians with experience from hepatology and clinical studies. The members will be independent of the sponsor and will not be investigators or collaborators in the current study.

### Interim analyses {21b}

There will be no interim analysis or stopping guidelines, except what is part of usual practice if any severe AEs occur (see below).

### Adverse event reporting and harms {22}

HCV assessment and treatment in this study will follow current HCV treatment recommendations, ensuring that the stage of liver disease, renal function, co-infections, and potential drug–drug interactions are assessed before initiation of treatment. Reported DAA side effects are generally mild and transient, including nausea, headache, and fatigue. Such mild AEs will not be collected systematically nor reported in the trial publication.

Despite a very favorable safety profile, it is essential to note that HCV protease inhibitors are contraindicated in decompensated cirrhosis (Child Pugh class B or C) due to the risk of fatal hepatotoxicity and should be used under close surveillance in compensated cirrhosis (Child Pugh class A). Although the intensity of follow-up will vary considerably in this trial, the preferred use of pan-genotypic treatment without a protease-inhibitor (sofosbuvir/velpatasvir) will necessarily eliminate these risks. Further, there is a slight risk of reactivation of hepatitis B virus (HBV) infection if DAAs are administered to patients with untreated HBV co-infection. Thus, to minimize this risk, HBV serology will be systematically followed up for individuals who are discharged before HBV status is present. Treatment with a nucleoside inhibitor (tenofovir) will be provided during the course of DAA treatment for all individuals who are diagnosed with HBV co-infection.

All severe AEs occurring during the course of the study will be followed up prospectively for each participant by the treating physician in accordance with usual clinical practice. If detected, events will be followed until resolution, unless the event is considered to be unlikely to resolve due to the underlying disease. Every effort will be made to obtain a resolution for all events, even if the events continue after discontinuation/study completion. All severe AEs will be reported and documented, and the investigators will perform an evaluation with respect to causality and expectedness. All severe AEs will be reported in the trial publication.

HCV replicates at high rates, allowing for rapid response to selective immune or drug-induced pressure that may select for resistance-associated substitutions in the viral genome. Consequently, including individuals with reduced adherence to treatment may increase the risk for the development of antiviral resistance among study participants. This study will provide unique opportunities to explore the prevalence and range of baseline resistance-associated substitutions and the extent to which they influence DAA treatment among PWID in real-world clinical practice.

### Frequency and plans for auditing trial conduct {23}

The data monitoring committee will meet every 6 months. The committee will report any severe AEs independent from the sponsor or investigators.

### Plans for communicating important protocol amendments to relevant parties (e.g. trial participants, ethical committees) {25}

Any important protocol modifications will be communicated routinely to the sponsor and the Regional Committee for Medical Research Ethics, as well as to the primary investigators, physicians, and nurses at the participating wards.

## Dissemination policy

### Plans for investigators and sponsor to communicate trial results {31a}

A detailed publication and dissemination plan will be developed well in advance of data extraction and analyses. The results will be published in peer-reviewed scientific journals; abstracts will be submitted for the most important international conferences in the field. The findings will also be communicated in relevant national conferences, to policymakers at all levels, and various media channels. The results will also be communicated to relevant user communities via the user representative of the trial steering committee.

### Plans to give access to the full protocol, participant-level data, and statistical code {31c}

The corresponding author can be contacted for access to other data or documentation.

## Discussion

A key obstacle to effective HCV care among PWID and global HCV elimination is the lack of treatment models within specialist healthcare adapted to marginalized individuals. This study aims to assess the efficacy of opportunistic treatment of HCV infection in PWID admitted for inpatient care in departments of internal medicine, addiction medicine, and psychiatry. The intervention will be tested at the level of care delivery, in a pragmatic clinical trial employing a stepped wedge cluster randomized design. Representing a novel treatment model suited to reach and engage PWID in HCV care, this study could inform HCV elimination efforts locally and internationally. If the model is found to be efficacious and feasible, it should be considered for broader implementation, replacing the current referral-based standard of care.

The study involves important limitations and challenges, as well as several strengths. The requirement for informed consent may introduce a selection effect that could compromise the generalizability that is central to the pragmatic trial approach. Individuals may be lost from inclusion due to psychosocial barriers (e.g. psychosis, drug influence, or reduced consciousness) or logistical barriers (e.g. failure to draw blood or early discharge before HCV RNA result is present) that are relatively specific to the PWID population. As a result, the most marginalized individuals may be excluded, favoring a study population where individuals with a higher level of social functioning are over-represented. To explore the significance of this bias, we will attempt to include individuals with early discharge for a sensitivity analysis. To compensate for logistical screening barriers, the use of point-of-care HCV RNA testing was considered. However, point-of-care HCV RNA platforms are not generally available in our region; introducing this element would therefore represent an important breach with the pragmatic clinical trial principles.

The complex multi-component study intervention may pose another challenge to the interpretation of the results. If the intervention were found to be effective, a timely question would be which component of the intervention is responsible for the effect. Although we cannot break down the effects of these separate components, we believe it is the totality of a potentially feasible and disseminative intervention that is most relevant for informing clinical practice in this field.

Although flexible intervention delivery and intention-to-treat analysis are considered hallmarks of the pragmatic clinical trial, it may also introduce methodological challenges. For instance, HCV treatment for PWID in Norway is currently provided at several primary care-based low-threshold settings. Therefore, we may experience that individuals included in control periods are successfully engaged in low-threshold HCV care in the meantime while waiting for an appointment at the outpatient clinic. Analysis according to an intention-to-treat principle may in such situations lead to a higher proportion of individuals in the control group accomplishing the primary outcome, and subsequently, less difference in effect between intervention and control groups, than estimated and expected in regions without proper access to low-threshold clinics. This may, in turn, lead to a generalizability problem.

Similarly, as a consequence of increasing uptake of treatment among PWID in the wake of the recent Norwegian HCV elimination strategy, HCV RNA prevalence among PWID is declining in Norway. Hence, the inclusion of study participants may be increasingly difficult with time. On the other hand, HCV awareness and screening routines in the participating clusters may improve due to the implementation of the study, thereby balancing out this bias. Thus, calendar time is a potential confounder associated with the exposure to the intervention and will be adjusted for in the statistical model. In order to explore these aspects further and attempt to capture the ongoing dynamics of local HCV epidemiology, we plan to perform additional subgroup analyses and also time-dependent analyses with time-to-event as outcomes.

Another key challenge inherent to the pragmatic clinical trial may be evident in that physicians may find it more appropriate to handle the patients in conflict with the protocol. For instance, when a patient is identified with advanced liver disease, the physician could find it more appropriate to start treatment immediately instead of referring to delayed outpatient care. Conversely, we also expect to include patients with a level of social functioning considered too low to justify immediate treatment initiation. Such protocol violations are in the nature of pragmatic trials and will probably not induce a significant bias as long as they are evenly distributed across the clusters.

As the study will include many marginalized individuals with a reduced ability to adhere to treatment, a critical challenge will be to ensure the retention of patients in treatment and post-treatment follow-up. Again, we aim to determine the best standards of care and examine if the intervention will work under usual conditions and therefore do not suggest including interventions that are not likely to be available in clinical practice, e.g. electronic reminders, systematic use of directly observed therapy, or economic incentives. However, great efforts will be made to avoid compromising the established standards of post-treatment care, involving hepatocellular carcinoma surveillance for patients with liver cirrhosis and HCV RNA surveillance for individuals with injecting risk behaviors.

Despite its potential pitfalls, we consider the pragmatic stepped wedge design highly appropriate for political, logistical, and statistical reasons. A key novelty of this trial is its potential to include a relatively unselected population of PWID, including marginalized individuals with decreased ability to benefit from existing treatment models and take part in ordinary clinical trials. This is particularly important in the field of HCV care, where existing knowledge mostly has been derived from larger treatment trials of highly selected individuals or from smaller cohort studies of PWID, with a palpable scarcity of randomized controlled trials. The pragmatic approach without a separately constructed research infrastructure allows for measurement of the benefit of an intervention under generalizable real-life clinical conditions with the aim to inform decision-makers and improve the health of an underprivileged group.

From a strategic and fairness perspective, it is a clear advantage that the design inherently ensures that all participating clusters will provide the intervention. If the intervention proves effective, it would be more convenient for stakeholders and decision-makers to continue an intervention that has already been implemented than to establish the model from scratch. Logistically, the stepped wedge design allows for a gradual implementation of the intervention with possibilities for problem-solving and practical adjustments underway. As such, the design naturally incorporates a pilot phase in the first part of the study period. In addition, by utilizing resources already paid for by the hospitals, the study can include many participants over a relatively short time duration at a lower cost than studies utilizing traditional randomized controlled trial designs with an external study organization. We also hope that increasing local HCV awareness during the study period will generate positive ripple effects more broadly and contribute to HCV elimination in Norway. The fact that local physicians will be increasingly involved in HCV treatment represents a valuable educational aspect of the study.

As discussed earlier, the design also provides statistical advantages over a traditional parallel-group cluster randomization design. Assuming a large intra-cluster correlation coefficient, the stepped wedge design is considered robust with regards to statistical power, partly due to the fact that all clusters will act their own control. Moreover, with this design, we can compare exposed and unexposed periods within a cluster, allowing exploration of heterogeneity in treatment effects between clusters and also the impact of the intervention over time. The statistical analysis using a mixed logistic regression model is considered appropriate and recommended for this design. By virtue of being clinically relevant and easy to measure without the need for structured follow-up, we consider the chosen outcome measures appropriately pragmatic. Despite the expected loss to follow-up, the availability of high-quality health registries in Norway will ensure that relevant data for assessment of the outcomes are accessible.

Finally, the trial may also create opportunities to disseminate a relatively new and increasingly appreciated study design. By learning the pragmatic game, we hope to generate a platform to perform further studies of diverse models of care for various patient populations, inside or outside the hospital setting. In particular, we consider the pragmatic trial approach suitable for further research in marginalized populations of PWID.

In conclusion, the pragmatic stepped wedge cluster randomized trial OPPORTUNI-C aims to assess the efficacy of a novel model of HCV care, involving an opportunistic strategy with immediate assessment and treatment of HCV infection among PWID admitted for inpatient care in departments of internal medicine, addiction medicine, and psychiatry. If the model is shown to be efficacious and feasible, it should inform HCV elimination efforts internationally, encouraging an opportunistic test-and-treat approach to PWID with chronic HCV infection.

## Trial status

The study protocol was registered at clinicaltrials.gov (NCT04220645) on 7 January 2020. The first study participant was included on 1 October 2019. As per 9 March 2020, 70 participants have been included. The trial is ongoing and recruitment will be completed by the end of January 2021.

## Data Availability

No datasets are included in this manuscript. Any data required to support the study protocol will be supplied on request. The data generated from the trial will be made publicly available in accordance with the publication policies of the relevant journal, either in a public repository or available on request.

## Abbreviations

AE: Adverse events
HCV: Hepatitis C virus
DAAs: Direct-acting antivirals
PWID: People who inject drugs
HBV: Hepatitis B virus
